# CD123-Targeted Nano-Curcumin Molecule Enhances Cytotoxic Efficacy in Leukemic Stem Cells

**DOI:** 10.3390/nano11112974

**Published:** 2021-11-05

**Authors:** Wariya Nirachonkul, Siriporn Ogonoki, Tarika Thumvijit, Supanimit Chiampanichayakul, Pawaret Panyajai, Songyot Anuchapreeda, Singkome Tima, Sawitree Chiampanichayakul

**Affiliations:** 1 Department of Medical Technology, Faculty of Associated Medical Sciences, Chiang Mai University, Chiang Mai 50200, Thailand; wariya_n@cmu.ac.th (W.N.); pawaret_panyajai@cmu.ac.th (P.P.); songyot.anuch@cmu.ac.th (S.A.); singkome.tima@cmu.ac.th (S.T.); 2 Department of Pharmaceutical Sciences, Faculty of Pharmacy, Chiang Mai University, Chiang Mai 50200, Thailand; siriporn.okonogi@cmu.ac.th; 3 Research Center of Pharmaceutical Nanotechnology, Faculty Chiang Mai University, Chiang Mai 50200, Thailand; 4 Department of Radiologic Technology, Faculty of Associated Medical Sciences, Chiang Mai University, Chiang Mai 50200, Thailand; tarika.thumvijit@cmu.ac.th; 5 Cancer Research Unit of Associated Medical Sciences (AMS CRU), Faculty of Associated Medical Sciences, Chiang Mai University, Chiang Mai 50200, Thailand; 6 Department of Chemistry, Faculty of Science, Naresuan University, Phitsanulok 65000, Thailand; supanimitc@nu.ac.th

**Keywords:** anti-CD123, leukemic stem cells, curcumin, acute myeloblastic leukemia, nanoparticles

## Abstract

Acute myeloblastic leukemia (AML) is a disease with a high rate of relapse and drug resistance due to the remaining leukemic stem cells (LSCs). Therefore, LSCs are specific targets for the treatment of leukemia. CD123 is specifically expressed on LSCs and performs as a specific marker. Curcumin is the main active compound of a natural product with low toxicity for humans. It has been reported to inhibit leukemic cell growth. However, curcumin is practically insoluble in water and has low bioavailability. In this study, we aimed to formulate curcumin nanoparticles and conjugate with the anti-CD123 to overcome the low water solubility and improve the targeting of LSCs. The cytotoxicity of both curcumin-loaded PLGA/poloxamer nanoparticles (Cur-NPs) and anti-CD123-curcumin-loaded PLGA/poloxamer nanoparticles (anti-CD123-Cur-NPs) were examined in KG-1a cells. The results showed that Cur-NPs and Cur-NPs-CD123 exhibited cytotoxic effects on KG-1a cells with the IC_50_ values of 74.20 ± 6.71 and 41.45 ± 5.49 µM, respectively. Moreover, anti-CD123-Cur-NPs induced higher apoptosis than Cur-NPs. The higher uptake of anti-CD123-Cur-NPs in KG-1a cells was confirmed by using flow cytometry. In conclusion, the anti-CD123-Cur-NPs formulation improved curcumin’s bioavailability and specific targeting of LSCs, suggesting that it is a promising drug delivery system for improving the therapeutic efficacy against AML.

## 1. Introduction

Acute myeloblastic leukemia (AML) is an aggressive and heterogeneous hematological malignancy that affects myeloid cells in the bone marrow and blood [1]. AML can be diagnosed in both adults and children, but most cases occur in adults [2]. In a previous report, about 50% of AML patients achieved complete remission (CR) with intensive treatment. However, approximately two-thirds of AML patients relapse after initial treatment, and most relapses occur within the first 18 months [3,4]. Recently, many studies have reported one major mechanism of AML relapse that is involved in the presence of clones known as leukemic stem cells (LSCs) [5]. LSCs are a minor cell population that originate from the malignant transformation of normal hematopoietic stem cells (HSCs). Similarly to HSCs, the LSCs possess capacities for self-renewal, proliferation, and differentiation. Interestingly, the prolonged residence of LSC populations in the quiescent (G_0_) stage and the highly expressed P-glycoprotein drug transporter have been shown to allow them to evade chemotherapeutic drugs. Therefore, LSCs are considered to be the root of chemotherapeutic drug resistance and disease relapse in AML.

As mentioned previously, the elimination of LSCs is the goal of AML treatment. Therefore, the identification of markers that are specific to LSCs and targeting to eradicate LSCs will be challenging. CD123, an interleukin-3 receptor alpha chain, plays an important role in the regulation of hematopoietic cell production [6]. It is widely overexpressed in various hematological malignancies, including AML. Importantly, CD123 is overexpressed in leukemic stem cells (LSCs) and leukemic blasts, but not in normal stem cells. CD123 thus represents a promising target molecule for drug delivery in order to specifically eradicate LSCs. Therefore, CD123, an established LSC marker, was a focus in this study.

Although chemotherapy is a major strategy for leukemia treatment, it is nonspecific to leukemic cells, and administration of high doses of chemotherapeutic drugs can cause adverse effects. However, natural products from medicinal plants are alternative choices for leukemia treatment. Curcumin (Cur) is a natural polyphenol constituent of turmeric (*Curcuma longa* Linn.) that exhibits potential medicinal uses against cancer, inflammation, oxidative stress, and microbial infection [7,8,9,10]. In a previous study, Anuchapreeda S. et al. demonstrated that curcumin showed a cytotoxic effect, induced cell death in human leukemic cell lines [11], and reversed MDR activity [12]. Recently, our group also reported that curcumin exhibited cytotoxicity in a KG-1a leukemic stem cell line and decreased the WT1 and FLT3 protein expression involved in cell proliferation [13]. All of these studies suggested that, as a product from a medicinal plant, curcumin is a good candidate for various cancer treatments, including leukemia treatment [11,12,13]. Several reports have shown the safety of curcumin in humans. Curcumin has been demonstrated as not toxic to humans in quantities up to 8000 mg/day when orally taken for 3 months [14]. Furthermore, curcumin did not show toxicity in humans after consumption at a high dose (12 g/day) [15]. Unfortunately, the efficacy of curcumin is limited because of its poor bioavailability, low water solubility, and rapid clearance [16,17]. The highest serum concentration of curcumin was found after oral administration for 1 to 2 h and gradually decreased within 12 h [14]. All of these limitations present a major barrier to practical use and clinical application. Many studies have found ways to solve these problems, including the synthesis of curcumin analogs [18,19] or the development of drug delivery systems (DDSs) for curcumin, such as trapping curcumin in nanoparticles [20,21].

Drug delivery systems (DDSs) via nanocarriers are currently popular approaches for improving drug dissolution and carrying hydrophobic drugs to target cells. With various kinds of nanocarriers, poly(DL-lactide-co-glycolic acid) is considered to be a promising carrier in drug delivery. PLGA, a copolymer of cyclin dimers (1,4-dioxane-2, 5-diones) of glycolic acid and lactic acid, has good biocompatibility, biodegradability, and stability in body fluids [22]. Many studies have been demonstrated the efficacy of Cur-loaded PLGA NPs such as improved bioavailability and insolubility of Cur [23]. Therefore, trapping hydrophobic drugs or active compounds of medicinal plants into PLGA polymeric nanocarriers is a promising approach to drug delivery. In addition, antibody-targeted drug delivery is a strategy that can deliver drugs to cancer cells. Thus, the encapsulation of curcumin into nanoparticles to form nanocurcumin and then conjugation of nanocurcumin with antibody specific LSCs is a promising strategy for leukemia treatment. However, it requires a specific surface marker that will enhance curcumin-loaded nanoparticles in cancer cells. As mentioned previously, CD123 is a promising biomarker that can be used as a valuable target in treatment. Therefore, to target LSCs with nanotechnology, a targeting ligand such as CD123 antibody can be used to conjugate with nanoparticles. 

In the present study, we aimed to encapsulate Cur into PLGA/poloxamer nanoparticles (Cur-NPs) and conjugate CD123 antibody to PLGA/poloxamer NPs to formulate anti-CD123–curcumin-loaded nanoparticles (anti-CD123-Cur-NPs) for targeting CD123 on LSCs. Then, the established Cur-NPs and anti-CD123-Cur-NPs were characterized for particle size, morphology, and entrapment efficiency. Furthermore, we investigated the cellular uptake, cytotoxic effect, and apoptosis induction of anti-CD123-Cur-NPs on KG-1a leukemic stem cells.

## 2. Materials and Methods

### 2.1. Cell Line and Reagents

The KG-1a cell line (acute myeloblastic leukemic stem cell line; ATCC^®^ CCL-246.1™) was used as a leukemic stem cell model. The KG-1a cells were cultured in IMDM medium supplemented with 20% fetal bovine serum (FBS), 1 mM L-glutamine, 100 U/mL penicillin, and 100 µg/mL streptomycin in a humidified incubator with an atmosphere comprising 5% CO_2_ at 37 °C.

Poly(DL-lactide-co-glycolide) (PLGA; lactide to glycolide ratio: 50:50) and Pluronic^®^ F127 (Poloxamer 407) were purchased from Sigma-Aldrich (St. Louis, MO, USA). The 1-ethyl-3-(3-dimethylaminopropyl) carbodiimide hydrochloride (EDC), N-hydroxysuccinimide (NHS), Iscove’s modified Dulbecco’s medium (IMDM), penicillin–streptomycin, and L-glutamine were obtained from Thermo Fisher Scientific (Waltham, MA, US). Fetal bovine serum (FBS) was purchased from Capricorn Scientific (HE, Germany). Crude curcuminoid mixtures were purchased from Thai-China Flavours and Fragrances (TCFF) (Nonthaburi, Thailand). Purified anti-human CD123 monoclonal antibody was purchased from Elabscience (Houston, TX, USA).

### 2.2. Analysis of Surface CD123 Expression through Flow Cytometry

KG-1a cells (5 × 10^5^) were resuspended in PBS containing 0.1% (*w*/*v*) BSA and incubated with PE-labeled CD123 antibody (Houston, TX, US) for 30 min at 4 °C. Subsequently, the cells were washed with cold PBS containing 0.1% (*w*/*v*) BSA and further collected through centrifugation. Then, the stained cells were resuspended and analyzed through FACSCalibur flow cytometer (BD Biosciences, San Jose, CA, USA).

### 2.3. Preparation of Curcumin-Loaded PLGA/Poloxamer Nanoparticles (Cur-NPs) with or without the Targeting Ab 

#### 2.3.1. Preparation of Curcumin-Loaded PLGA/Poloxamer Nanoparticles (Cur-NPs)

For the preparation of Cur-NPs, the terminal -OH groups of poloxamer were initially modified to -COOH groups prior to the antibody conjugation step [24,25]. Briefly, 3 g of poloxamer (Pluronic^®^ F127) was dissolved in 14 mL of tetrahydrofuran (THF), followed by stirring. The 4-Dimethylaminopyridine (DMAP) (Sigma-Aldrich Inc., St. Louis, MO, USA), triethylamine (Sigma-Aldrich Inc., St. Louis, MO, USA), and succinic anhydride (Sigma-Aldrich Inc., St. Louis, MO, USA) were mixed and stirred for 48 h at room temperature. The solution was dried and dissolved in 20 mL of chloroform (Thermo Fisher Scientific Inc., Waltham, MA, USA). Afterwards, excess succinic anhydride was removed by using a 0.45 µm PTFE membrane syringe filter (Thermo Fisher Scientific Inc., Waltham, MA, USA). Subsequently, carboxylated poloxamer was precipitated with ice-cold petroleum ether for 48 h. Finally, the precipitate was collected and dried. The carboxylated poloxamer product was identified with a Fourier-transform infrared spectrophotometer using Thermo Nicolet Nexus 470 FT-IR spectrometer (Nicolet, WI, USA).

Preparation of curcumin-loaded PLGA/poloxamer NPs was performed through the preparation of a PLGA–curcumin solution containing 10 mg of PLGA and 100 μg of curcumin in 1.5 mL of acetone. Then, the PLGA–curcumin solution was added dropwise into a 10 mg/mL poloxamer solution under stirring. This Cur-NP solution was then centrifuged by using an Amicon^®^ filtration device (Sigma-Aldrich Inc., St. Louis, MO, USA) at 4400 rpm for 50 min at 4 °C. The resultant Cur-NPs were resuspended in 1 mL of sterile deionized (DI) water. 

#### 2.3.2. Conjugation of CD123 Antibody to Cur-NPs (Anti-CD123-Cur-NPs) 

In brief, Cur-NPs were activated with 50 mmol/L of NHS and 100 mmol/L of EDC with pH 5.8 for 1 h. Subsequently, this solution was centrifuged at 12,000 rpm for 15 min, and the supernatant was removed. Finally, CD123 antibody was conjugated to the Cur-NPs with the carbodiimide reaction [26]. The amino group of CD123 antibody reacted with the carboxylated poloxamer on the PLGA/poloxamer NPs. CD123 antibody (5 µL) was added to the Cur-NPs in 800 µL of DI water and 200 µL of PBS at pH 7.4. The mixture was gently stirred overnight. Free Abs were removed through centrifugation at 12,000 rpm for 15 min, and anti-CD123-Cur-NPs were obtained by washing with PBS at pH 7.4. The resultant anti-CD123-Cur-NPs were resuspended in PBS at pH 7.4 for further study. 

The quantification of CD123 antibody on NPs was determined by the subtracting the protein concentration of supernatant after conjugation from the amount of protein at the start before conjugation [27]. The concentration of protein was determined by a BCA assay. A standard curve was prepared from bovine serum albumin (2 mg/mL). 

### 2.4. NP Characterization

The properties of the NPs, including the mean particle size, polydispersity index (PdI), zeta potential (ZP), entrapment efficiency (EE), and loading capacity (LC), were determined. The mean particle size, PdI, and ZP were characterized through photon correlation spectroscopy (PCS) (Zetasizer, Malvern Instrument, Worcestershire, UK). Fourier-transform infrared (FTIR) spectrometry using Thermo Nicolet Nexus 470 FT-IR spectrometer (Nicolet, WI, USA) was performed to characterize the conjugation of anti-CD123-Cur-NPs. The morphology and size distribution of NPs were investigated using a transmission electron microscope (TEM) (JEM2200FS, JEOL, Tokyo, Japan). 

The loading of curcumin in the NPs was quantified by using a simple colorimetric assay. Briefly, the Cur-NPs were lysed in HBSE-TX 100 (10 mM HEPES, 140 mM NaCl, 4 mM, and 1% TX-100). The curcumin loading in nanoparticles was then calculated and compared to the standard curve of curcumin in HBSE-TX 100. The standard curve of curcumin was obtained by measuring the absorbance of the curcumin solution (0–250 µM) in the HBSE-TX 100. The absorbance of curcumin in the HBSE-TX 100 was measured by using a microplate reader (M965+ microplate reader, Metertech, Taipei, Taiwan) at 450 nm. 

The EE and LC were calculated by using the following equations.

(1) $$\mathrm{EE}\;\left(\%\right)=\frac{\mathrm{Amount}\;\mathrm{of}\;\mathrm{curcumin}\;\mathrm{in}\;\mathrm{NPs}}{\mathrm{Total}\;\mathrm{amount}\;\mathrm{of}\;\mathrm{curcumin}\;\mathrm{used}}\times 100\;$$

(2) $$\mathrm{LC}\;\left(\%\right)=\frac{\mathrm{Amount}\;\mathrm{of}\;\mathrm{curcumin}\;\mathrm{in}\;\mathrm{NPs}}{\mathrm{Total}\;\mathrm{amount}\;\mathrm{of}\;\mathrm{polymer}\;\mathrm{used}}\times 100$$

### 2.5. Cellular Uptake of Anti-CD123-Cur-NPs into the CD123-Expressing KG-1a Cell Line

The cellular uptake of anti-CD123-Cur-NPs into KG-1a cells was investigated by using flow cytometry. KG-1a cells (1.5 × 10^6^ cells/mL) were plated on a 6-well plate. Then, the cells were treated with 6 µM of curcumin from the Cur-NPs and anti-CD123-Cur-NPs in 10% FCS-IMDM and incubated at 37 °C for 5, 30, and 60 min. Following treatment, the cells were harvested, washed twice, and resuspended with PBS at pH 7.4. Finally, the fluorescent signal from curcumin was measured through flow cytometry and analyzed with the FlowJo V10 program.

### 2.6. In Vitro Cytotoxicity Assay

The cytotoxicity of empty NPs, Cur-NPs, and anti-CD123-Cur-NPs against the KG-1a cell line was evaluated through a colorimetric MTT assay. Briefly, KG-1a cells (1.5 × 10^5^ cells/well) were seeded in 96-well plates and incubated at 37 °C with 5% CO_2_ for 24 h. The cells were then treated with various concentrations (0–100 µM) of curcumin from the Cur-NPs and anti-CD123-Cur-NPs and then further incubated for 48 h. Empty NPs were used for comparison. After removing the medium (100 µL), MTT dye solution (15 µL) was added and incubated for 4 h. The supernatant was then discarded, and DMSO (200 µL) was added to dissolve the formazan crystals. The optical density was measured by using a microplate reader at 578 nm with a reference wavelength of 630 nm.

### 2.7. Cytotoxicity of Peripheral Blood Mononuclear Cells (PBMCs)

PBMCs were isolated from whole blood using the Ficoll-Paque density centrifugation method. PBMCs (1.0 × 10^6^ cells/mL) were plated on a 96-well plate and incubated at 37 °C, 5% CO_2_ overnight. Various concentrations (3.125, 6.25, 12.5, 25, 50, and 100 µg/mL) of curcumin from Cur-NPs, and anti-CD123-Cur-NPs were added, then cells were incubated for 48 h. The cell survival rate was assessed utilizing the MTT colorimetric assay, as previously described.

### 2.8. Apoptosis Assay

For the apoptosis assay, the KG-1a cells were plated on a 6-well plate at density 6 × 10^5^ cells/well. Then, cells were treated with empty NPs, Cur-NPs, and anti-CD123-Cur-NPs for 48 h. Cells were stained with 5 µL Annexin V-FITC and 10 µL PI solution and incubated at room temperature for 15 min in the dark. Then, stained cells were analyzed by flow cytometry.

### 2.9. Statistical Analysis 

The experiments were performed independently in triplicate, and the results are represented as mean ± SD. Statistical differences between the means were determined by using one-way ANOVA. The statistical differences were considered significant at *p* < 0.05 and very significant at *p* < 0.01.

## 3. Results

### 3.1. NP Formulation

**Figure 1:**
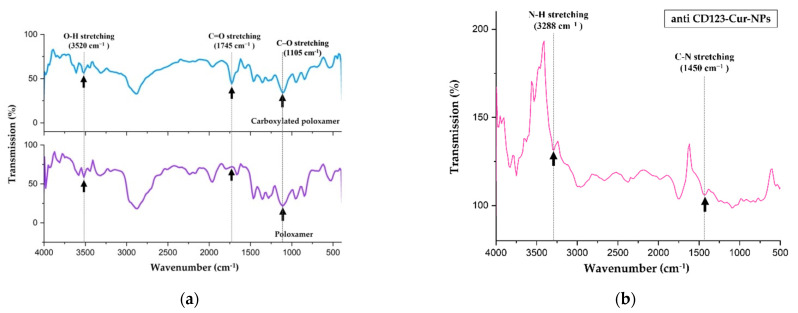
(**a**) FTIR spectra of the carboxylated poloxamer (**top**) and hydroxylated poloxamer (**bottom**); (**b**) FTIR spectrum of the anti-CD123-Cur-NP conjugate.

In order to prepare the antibody conjugates, the terminal hydroxyl (−OH) groups on poloxamer were initially modified into carboxyl (–COOH) groups, which are necessary for conjugation with the terminal amine group of the CD123 antibody. This successful conversion was confirmed by IR spectroscopic analysis. The FTIR spectrum showed a new absorption peak at 1745 cm^−1^, which corresponded to strong carbonyl (C=O) stretching vibrations of carboxylic (–COOH) groups (top IR spectrum in Figure 1a). This result suggested the formation of the carboxylated poloxamer. Then, the carboxylated poloxamer was reacted with PLGA to form NPs. Subsequently, the encapsulation of curcumin in the NPs produced curcumin-loaded PLGA/poloxamer nanoparticles (Cur-NPs). 

To study the targeting of leukemic-stem-cell-expressed CD123 (CD123-LSCs), the CD123 antibody was then conjugated to the carboxylated poloxamer of Cur-NPs. The structure of the anti-CD123-Cur-NP conjugate was further verified through FTIR spectroscopy. As shown in Figure 1b, the disappearances of carboxylic O–H stretching peak at 3520 cm^−1^ and carboxylic C–O stretching peak at 1105 cm^−1^ confirmed that the conjugation between the carboxylated poloxamer and CD123 was successful. Further confirmation is evidenced by the appearances of the new IR peaks related to the amide functional group. The diagnostic peaks at 3288 and 1450 cm^−1^ were attributed to the N–H and C–N stretching of amide, respectively. Certain identification of a carbonyl group of amide is difficult because several other absorption peaks can appear in the region 1650–1750 cm^−1^.

### 3.2. Characterization of Nanoparticles

In this study, the encapsulation of curcumin in nanoparticles was performed by using the nanoprecipitation technique [28]. PLGA and carboxylated poloxamer were chosen and applied to form NPs. Then, three different types of NPs were formulated, including PLGA/poloxamer nanoparticles without curcumin (empty NPs), curcumin-encapsulated PLGA/poloxamer nanoparticles (Cur-NPs), and curcumin-encapsulated PLGA/poloxamer nanoparticles conjugated with CD123 antibody (anti-CD123-Cur-NPs). 

**Figure 2:**
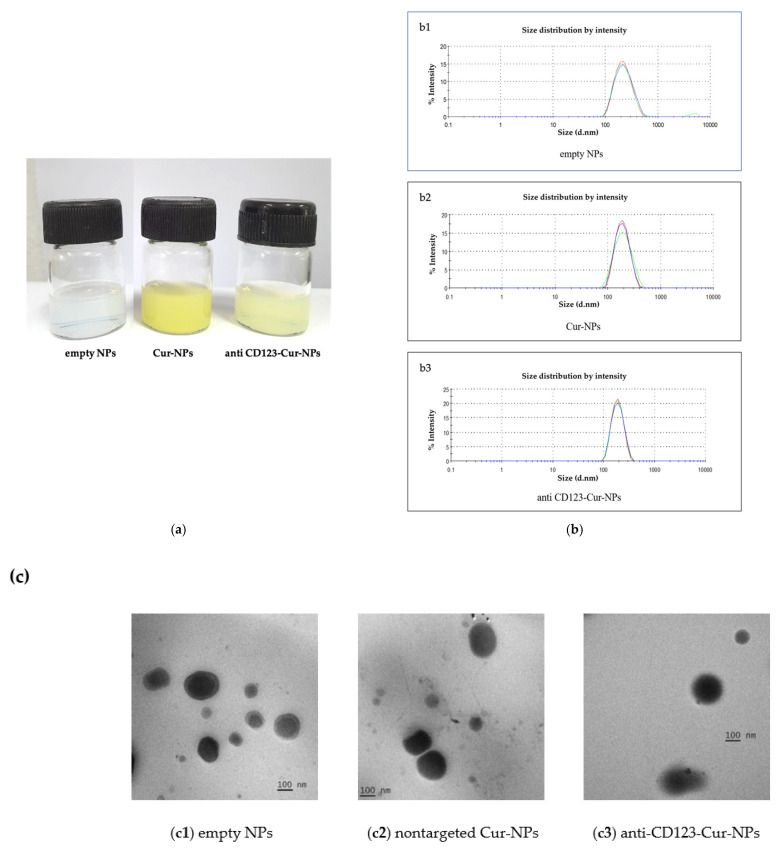
Characterization of nanoparticles. (**a**) Color of empty NPs, Cur-NPs, and anti-CD123-Cur-NPs. (**b**) Size measurement of the nanoparticles using dynamic light scattering (DLS); (**b1**) empty NPs, (**b2**) Cur-NPs, and (**b3**) anti-CD123-Cur-NPs. (**c**) TEM image of (**c1**) empty NPs, (**c2**) nontargeted Cur-NPs, and (**c3**) anti-CD123-Cur-NPs.

As shown in Figure 2a, the physical characterization of NPs demonstrated that the Cur-NPs were in a clear yellowish solution, whereas anti-CD123-Cur-NPs were in a slightly turbid yellowish solution. The empty NPs (without curcumin) were clear and colorless. The properties of the NPs were evaluated, and the data are summarized in Table 1. The DLS histogram showed that the average size of the NPs varied in the range of 181.27 ± 0.07 to 214.37 ± 3.23 nm in diameter, suggesting the sizes of the nanoparticles (Table 1 and Figure 2b). Moreover, the polydispersity index (PdI) of all NPs (between 0.06 ± 0.03 to 0.09 ± 0.05) was less than 0.3, indicating that the distribution of the NPs’ size was monodispersed [29]. The measurement of the zeta potential values revealed that the CD123 antibody’s conjugation to the NPs decreased the surface charge (–32.87 ± 1.63 and –46.21 ± 0.07 mV for nontargeted Cur-NPs and anti-CD123-Cur-NPs, respectively). The entrapment efficiency of curcumin in the Cur-NPs and anti-CD123-Cur-NPs was 88.64% and 77.56%, respectively (Table 1). The morphology of empty NPs, Cur-NPs, and anti-CD123-Cur-NPs was observed using a transmission electron microscope (TEM). As shown in Figure 2c, all NPs exhibited spherical morphology. The percentage of CD123 antibody on surface of NPs measured using the indirect method was 70.8%. 

### 3.3. Cellular Uptake of Anti-CD123-Cur-NPs

**Figure 3:**
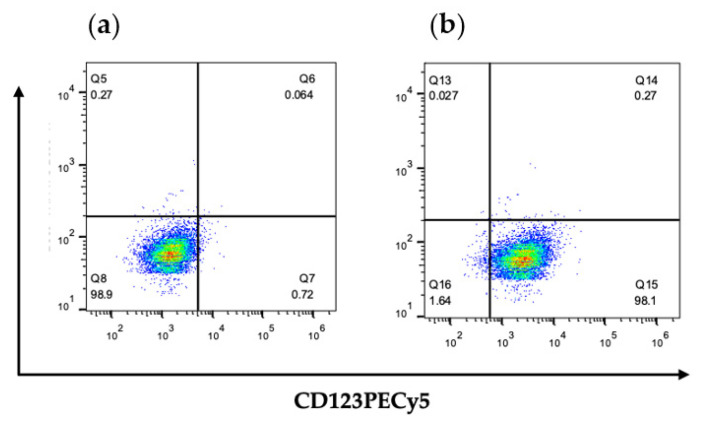
Surface expression of CD123 on KG-1a cells. Cells were stained with (**a**) isotype-matched control antibody or (**b**) CD123PECy5 antibody. Stained cells were assessed through flow cytometry.

**Figure 4:**
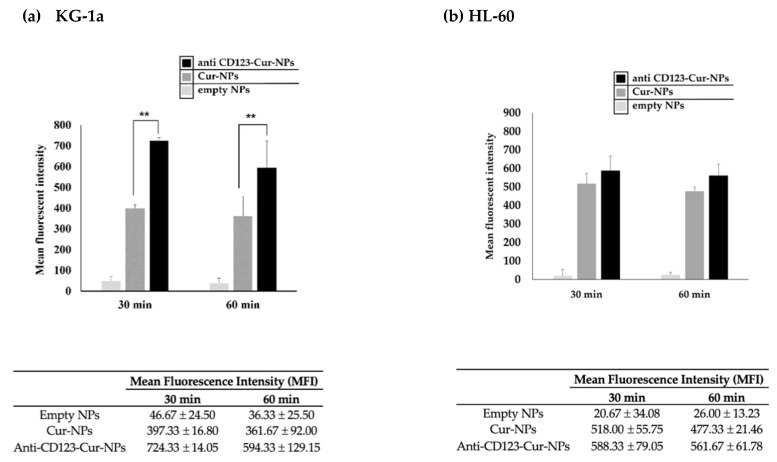
Cellular uptake of anti-CD123-Cur-NPs or nontargeted Cur-NPs in KG-1a and HL-60 leukemia cell lines. KG-1a (CD123+) (**a**) and HL-60 (CD123-) (**b**) cells were incubated with anti-CD123-Cur-NPs or Cur-NPs for 30 and 60 min, and fluorescent intensity was determined through flow cytometry. The experimental data were collected in three independent experiments, and the results were determined using one-way ANOVA. The statistical differences were considered significant at ** *p* < 0.01.

The efficacy of anti-CD123-Cur-NPs, a targeted drug delivery system, was determined by the cellular uptake of the NPs by CD123+ KG-1a and CD123 negative (CD123-) HL-60 cell lines that expressed CD123 molecules on their cell surface. Because curcumin exhibits strong fluorescence, curcumin was used as a fluorescent agent for monitoring the cellular uptake in this experiment. As shown in Figure 3, flow cytometry revealed that all KG-1a cells expressed CD123 molecules. This cell line was then used to study the CD123-specific targeting of NPs. The cellular uptake of nontargeted Cur-NPs and anti-CD123-Cur-NPs was assessed with flow cytometry. The fluorescence intensity exhibited high cellular uptake of anti-CD123-Cur-NPs by KG-1a cells. The result was significantly higher than that of the nontargeted Cur-NPs at 30 and 60 min (*p* < 0.01) (Figure 4). This result suggests that the anti-CD123-Cur-NPs were taken up by a CD123-dependent uptake mechanism, whereas the fluorescence intensity of Cur showed no difference between nontargeted Cur-NPs and anti-CD123-Cur-NPs in the CD123- HL-60 cell line.

### 3.4. Cytotoxicity Assay

**Figure 5:**
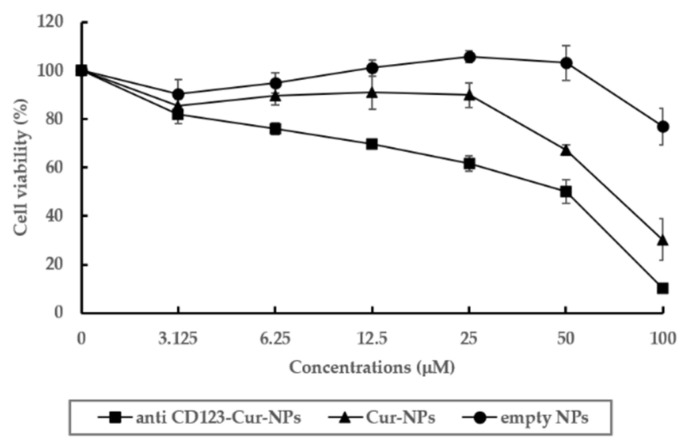
Cytotoxicity of NPs in KG-1a cells. Cell viability of KG-1a cells was assessed with an MTT assay. The data are shown as mean ± SD from three independent experiments.

The cytotoxicity of anti-CD123-Cur-NPs in KG-1a cells was compared to that of Cur-NPs and empty NPs. The anti-CD123-Cur-NPs, Cur-NPs, and empty NPs were added and cultured with KG-1a cells for 48 h. Then, an MTT assay was performed. As shown in Table 2, the Cur-NPs and anti-CD123-Cur-NPs showed cytotoxic effects on KG-1a cells with IC_50_ values of 74.20 ± 6.71 and 41.45 ± 5.49, respectively. In addition, the reduction by anti-CD123-Cur-NPs of KG-1a cells’ viability was dependent on the dose (Figure 5), whereas the empty NPs did not show cytotoxicity. From these results, it was suggested that anti-CD123-Cur-NPs were the best vehicle form for carrying curcumin to the target CD123-expressing KG-1a leukemic stem cells. In addition, the cytotoxicity of the anti-CD123-Cur-NPs corresponded to the mechanism of cellular uptake through a CD123-mediated process, while Cur-NPs were taken up by cells via a non-selective endocytic mechanism [30]. 

**Figure 6:**
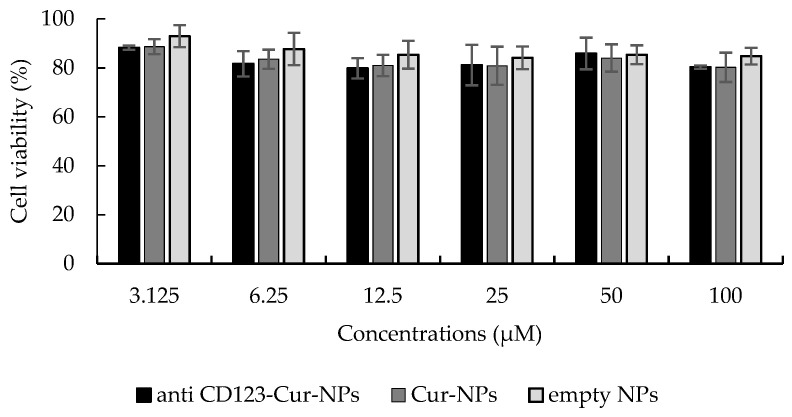
Cytotoxicity of NPs to peripheral blood mononuclear cells (PBMCs). PBMCs were treated with anti-CD123-Cur-NPs, Cur-NPs, and empty NPs for 48 h. The MTT assay determined the cell viability. Each bar represents the mean ± SD of three independent experiments performed in triplicate.

Moreover, the cytotoxic effect of NPs was evaluated in peripheral blood mononuclear cells (PBMCs), a representative normal blood cell model. The IC_50_ values of anti-CD123-Cur-NPs, Cur-NPs, and empty NPs in PBMCs were > 100 µg/mL (Figure 6). These results indicated that neither NPs had cytotoxic effect on PBMCs. 

### 3.5. Anti-CD123-Cur-NPs Induce Apoptosis of KG-1a Cell Line 

**Figure 7:**
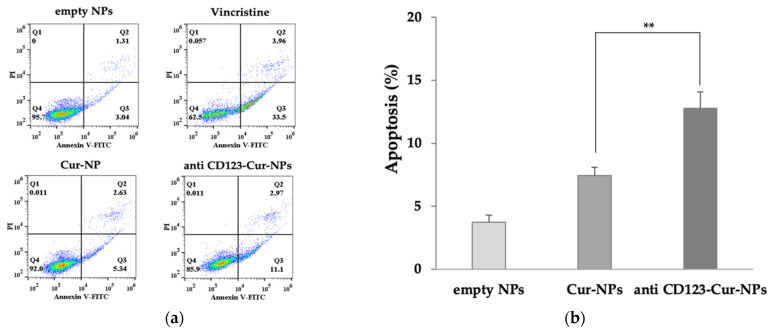
Apoptosis assay using flow cytometry after staining with double annexin V-FITC/propidium iodide (PI). KG-1a cells were treated with empty NPs, Cur-NPs, and anti-CD123-Cur-NPs. (**a**) Representative flow cytometry dot plot indicating the cell population in apoptotic and necrotic quadrants after treatment with different NPs. (**b**) The percentage of apoptotic cells was statistically compared. The data are shown as mean ± SD from three independent experiments. ** *p* < 0.05 between groups.

The percentage of apoptotic cells was measured after treatment with empty NPs, Cur-NPs, and anti-CD123-Cur-NPs. Vincristine was used as positive control. As shown in Figure 7, Cur-NPs and anti-CD123-Cur-NPs increased the fraction of apoptotic cells compared with empty NPs (7.43 ± 0.64% and 12.77 ± 1.30%, respectively). A comparison between the Cur-NPs and anti-CD123-Cur-NPs showed that the difference was significant (*p* < 0.05). This result indicated that anti-CD123-Cur-NPs improved the ability of curcumin to induce apoptosis by targeting the cells that express CD123 protein.

## 4. Discussion and Conclusions

AML is a type of blood cell malignancy that is characterized by uncontrolled proliferation and abnormal differentiation of myeloid progenitors. The standard treatment of AML is based on chemotherapy, which uses potent cytotoxic drugs to treat cancer. However, chemotherapy can induce relapse in two-thirds of AML patients after first-line treatment [3]. Moreover, approximately 10–40% of new AML patients who do not achieve complete remission are categorized as primary refractory or resistant patients [4]. As reported, LSCs are considered to be the cause of drug resistance and relapse [31,32,33]. Several studies reported that LSCs can regenerate AML in mouse models, in addition to initiating and maintaining AML [34,35,36]. LSCs also promote drug resistance because they remain in the quiescent stage (G_0_) of the cell cycle and overexpress P-glycoprotein, a multidrug resistance transporter. Therefore, the targeted elimination of LSCs is the goal of AML treatment. Moreover, the use of high doses of chemotherapeutic drugs to destroy LSCs can cause toxic effects on normal cells and leads to serious toxic and adverse effects. 

Recently, medicinal plants have been reported elsewhere as an alternative choice for the treatment of cancer. Curcumin (Cur) is a well-known major active compound that is present in the rhizomes of turmeric (*Curcuma longa* Linn.). Several studies have indicated that Cur has been shown to have immunomodulatory, anti-oxidant, anti-cancer, and anti-inflammatory activities [7,8,9,10]. It also has cytotoxic effects on cancer stem cells, including leukemic stem cells. Moreover, our previous study indicated that Cur exhibited an inhibitory effect on the proliferation of the KG-1a leukemic stem cell line due to its ability to inhibit the expression of the WT1 and CD34 proteins [13]. However, the major problem with Cur is its poor bioavailability. Thus, many studies have attempted to develop curcumin formulations with greater bioavailability. Nanotechnology is one of the strategies that have been investigated for this purpose. Many studies reported that curcumin nanoparticles possess significantly greater water solubility and bioavailability than free curcumin [37,38,39,40].

This led us to design and formulate our curcumin nanoparticles, which indeed exhibited cytotoxic activities in the KG-1a leukemic stem cell line. In the present study, curcumin was successfully incorporated into PLGA/poloxamer nanoparticles (Cur-NPs). It was shown that the encapsulation of Cur with PLGA/poloxamer reflected the improvement of curcumin’s solubility. Consistently with our results, Yallapu MM et al. showed an improved anti-cancer potential in terms of cell proliferation compared to free curcumin [39]. Chang PY et al. reported that curcumin-loaded nanoparticles induced apoptosis in cisplatin-resistant human oral cancer cells [41]. In addition, a previous study reported that curcumin-loaded PLGA nanoparticles are more potent in inducing cancer cell apoptosis. They enhanced cellular uptake and had increased bioavailability [42,43,44]. As mentioned above, various nanocurcumin including micelles and polymeric NPs have been reported. Moreover, Hu et al. applied a targeted drug delivery system by using a specific small peptide to carry GE11 peptide-conjugated nanoparticles that carried doxorubicin [45]. This approach showed an improved therapeutic efficacy against breast cancer, confirming the potential of targeted nanoparticle systems. 

Targeted drug delivery systems that use antibodies that recognized specific surface proteins expressed on LSCs are considered to be a potential strategy for cell-specific treatment. This strategy can also be used to overcome the toxicity of chemotherapeutic drugs in normal cells. An antibody-targeted drug delivery system requires a suitable cell surface marker that will enhance the nanoparticles to the site of disease. Recently, many surface antigens on LSCs, including CD96, TIM3, and CD123, were identified. In our approach, the CD123 interleukin-3 receptor alpha chain (IL-3Rα) was selected because it has been reported to be prominently expressed on CD34^+^CD38^–^ leukemic stem cells in leukemia but not on normal hematopoietic cells [46]. Therefore, CD123 is an important surface marker for the identification and targeting of LSCs [47,48]. As reported, leukemic stem cells and leukemic progenitor cells have been demonstrated to have overexpression of CD123 in comparison with normal HSCs [46,49]. Here, we demonstrated the high expression of CD123 in the KG-1a leukemic stem cell line, suggesting the importance of CD123 as a specific LSC marker.

In this study, we developed a targeted nanocurcumin, anti-CD123-Cur-NPs, and investigated it for anti-leukemia activity. To our knowledge, this is the first report on development and evaluation of CD123-targeted nanocurcumin for AML.

We successfully formulated anti-CD123-antibody-conjugated curcumin-encapsulated nanoparticles (anti-CD123-Cur-NPs) that could improve the targeting of LSCs. 

We used EDC-NHS carbodiimide reaction to conjugate CD123 antibodies to the curcumin-loaded PLGA/poloxamer nanoparticles. The particle size of anti-CD123-Cur-NPs was less than 200 nm and could avoid rapid renal clearance and phagocytosis by the reticuloendothelial (RE) system [50]. The morphology and size distribution of anti-CD123-Cur-NPs were confirmed by transmission electron microscopy. All NPs, including anti-CD123-Cur-NPs, showed spherical morphology. The entrapment efficiency (%EE) of anti-CD123-Cur-NPs was lower than Cur-NPs because of the conjugation step [26]. Next, we evaluated the anti-CD123-Cur-NPs for their targeting potential and cytotoxicity in KG-1a leukemic stem cell model. The cell viability in the presence of nontargeted Cur-NPs and anti-CD123-Cur-NPs in the KG-1a leukemic stem cell line was quantified with an MTT assay. The IC_50_ value clearly demonstrates that anti-CD123-Cur-NPs (IC_50_ = 41.45 ± 5.49) were more effective than Cur-NPs (IC_50_ = 74.20 ± 6.71) suggesting the relation of the anti-CD123 conjugated to the surface of the NPs. These results demonstrated that the targeting of KG-1a cells with curcumin and the induction of cytotoxic effects were mediated in a CD123-dependent manner. It has been reported that CD123 is actively internalized by the cells after antibody binding; in contrast, nontargeted Cur-NPs entered the cells through a passive uptake strategy [51]. This result is consistent with several previous studies on the cytotoxic effect of curcumin and nanocurcumin on cancer cells. The flow cytometry results further confirmed the high uptake of anti-CD123-Cur-NPs by the KG-1a cells in comparison with the Cur-NPs. These results indicated that the targeting specificity of the anti-CD123-Cur-NPs led to the improvement of the cytotoxic effects of curcumin on KG-1a cells. 

Furthermore, the cellular mechanism underlying the cytotoxicity of curcumin was also investigated. We performed Annexin V/PI staining and flow cytometry to evaluate the nanocurcumin on apoptosis. The percentage of apoptotic cells in Cur-NPs and anti-CD123-Cur-NPs was observed when compared with empty NPs. This may be explained by the cellular mechanism of cytotoxicity due to the action of curcumin. Previous reports showed that curcumin promoted apoptosis through the activation of caspase-9 and caspase−3 in a K562 leukemia cell line [52,53]. Another study found that curcumin activates both apoptosis and autophagy in K562 cells [54]. In addition, we found that KG-1a treatment with anti-CD123-Cur-NPs significantly enhanced the apoptotic cell number compared to Cur-NPs. This increased activity was the result of the combination action of curcumin and CD123 antibody. However, both Cur-NPs and anti-CD123-Cur-NPs did not show a cytotoxic effect on normal peripheral blood mononuclear cells (PBMCs). The flow cytometry results further confirmed the high uptake of anti-CD123-Cur-NPs by the KG-1a cells in comparison with the Cur-NPs. These results indicated that the targeting specificity of the anti-CD123-Cur-NPs led to the improvement of the cytotoxic effects of curcumin on KG-1a cells. 

In conclusion, our data demonstrate that the encapsulation of Cur with PLGA/poloxamer and conjugated anti-CD123 antibody on the surface of the nanoparticles can serve as a potential vehicle that improves the bioavailability and specific delivery of Cur to KG-1a leukemic stem cells that express CD123.

## Figures and Tables

**Table 1:** Characteristics of the nanoparticles.

Nanoparticles	Size (nm)	PdI	ZP (mV)	EE (%)	LC (%*w*/*w*)
Empty NPs	214.37 ± 3.23	0.09 ± 0.05	−30.32 ± 3.10	-	-
Cur-NPs	183.78 ± 4.38	0.06 ± 0.03	−32.87 ± 1.63	88.64	0.42
Anti-CD123-Cur-NPs	181.27 ± 0.07	0.07 ± 0.03	−46.21 ± 0.07	77.56	0.25

The data are represented as mean ± SD from three independent experiments.

**Table 2:** Cytotoxicity of curcumin nanoparticles and empty nanoparticles in KG-1a cells.

Sample	IC_50_ (μM)
Anti-CD123-Cur-NPs	41.45 ± 5.49 **
Cur-NPs	74.20 ± 6.71
Empty NPs	>100

The data are represented as mean ± SD from three independent experiments. ** *p* < 0.01 compared with Cur-NPs.

## Data Availability

Data is contained within the article.
